# Design and Nutrient Analysis of a Carotenoid-Rich Food Product to Address Vitamin A and Protein Deficiency

**DOI:** 10.3390/foods10051019

**Published:** 2021-05-07

**Authors:** Kristina Lewandowski, Xiaoyu Zhang, Micala Hayes, Mario G. Ferruzzi, Chad M. Paton

**Affiliations:** 1 Department of Food Science & Technology, University of Georgia, Athens, GA 30602, USA; kristina.lewandowski1@gmail.com (K.L.); zhangxiaoyu_1017@sina.com (X.Z.); 2 Plants for Human Health Institute, North Carolina State University, Kannapolis, NC 28081, USA; mdhayes@ncsu.edu (M.H.); mferruz@ncsu.edu (M.G.F.); 3 Department of Foods & Nutrition, University of Georgia, Athens, GA 30602, USA

## Abstract

Worldwide undernutrition affects over 820 million individuals and is the underlying cause of over 50% of all childhood deaths. Sweet potatoes have been promoted to address vitamin A (vitA) deficiency, with a single, orange-fleshed sweet potato (OFSP) providing enough vitA, as β-carotene, to meet daily needs. However, the bioavailability of β-carotene is dependent on the presence of dietary fat, which is not provided by OFSP, and it lacks some essential amino acids. Therefore, in an attempt to create a food product that meets daily vitA requirements with adequate bioavailability and complete protein, we designed and assessed a sweet potato, peanut paste, and legume product. The final food product formulation, developed through computer modeling, resulted in a 65/5/35 (*w/w/w*) formulation in a 250 g serving and ~330 kcal. We then confirmed the nutrient content of macronutrients, and essential amino acids, zinc, and iron contents. Total β-carotene was assessed by HPLC and was lower than predicted through computer modeling, likely due to losses through thermal processing and/or degradation from storage. The results of this project indicate that the three ingredients can be combined into a single 250 g food product to provide >300 kcal energy, complete protein, and micronutrients in a more bioavailable form.

## 1. Introduction

Undernutrition is a worldwide public health concern affecting over 800 million individuals [1]. Sub-Saharan Africa (SSA) is one region where undernutrition is most prevalent, affecting almost one-quarter of the population. Additionally, the number of individuals who are undernourished has risen from 175 million in 2000 to 224 million in 2017. Undernutrition is a general term that can present as macronutrient deficiencies (total energy, protein, and fat) or micronutrient deficiencies (vitamins and minerals). Macronutrient deficiencies of total energy and protein are common in SSA, contributing to high rates of stunting and wasting, affecting 40% and 10% of children, respectively. Micronutrient deficiencies are also common, contributing to stunted growth and delayed mental development in children, as well as impaired immune response, and even death [2].

The major causes of vitamin A deficiency (VAD) are low intake of vitamin-A-rich foods combined with poor absorption and utilization in the body [3]. Food security crops in SSA include cereal grains and tubers, which are rich in energy but low in vitamin A [4]. Additionally, the vitamin A form that is found in plant-based products, predominantly existing as provitamin A carotenoids such as β-carotene, is less bioavailable than the form in animal-based food products and is dependent on a multitude of factors, most importantly the presence of dietary fat in the body [5]. Thus, when addressing VAD in these regions, both the availability of vitamin-A-rich foods and the bioavailability of the provitamin A carotenoids are important factors to consider.

Currently, there are various methods in place to address VAD, including the use of orange-fleshed sweet potatoes (OFSPs) [3]. OFSPs are rich in β-carotene, the main provitamin A carotenoid form, which, in the body, can be converted to the active form of vitamin A [6]. A single OFSP can provide sufficient amounts of provitamin A carotenoids to meet the daily vitamin A needs of individuals and have already shown positive results in increasing vitamin A levels in children [7,8]. Despite their ability to provide a sufficient amount of β-carotene, the bioavailability is hindered by the lack of fat. Muzhingi et al. demonstrated that inclusion of a fat source along with OFSP significantly improved the absorption and metabolism of β-carotene and subsequent vitA status [9].

Conversion of β-carotene to retinol can occur in the liver as well as in the proximal small intestine where absorption is highest [10]. Additionally, we and others have shown that dietary lipid composition affects the rate of TG absorption from the gut, with monounsaturated lipid (MUFA) absorbed earlier than saturated fat (SFA) [11,12]. Consistent with these findings, carotenoid absorption from vegetables was reported to be enhanced by co-consumption of a dietary lipid source rich in MUFA [13] and this effect was driven by modification of chylomicron secretion [14]. It is possible that such enhancement would impact β-carotene absorption and metabolism from OFSP products. Tawanda et al. demonstrated that OFSP consumption along with peanut paste improved vitA status better than OFSP alone or with SFA [9]. Peanuts are enriched in MUFA and are a logical choice for inclusion in a food product that is intended for improving vitA status with OFSP.

When considering nutritional approaches to combat vitA deficiency, it is important to take a systemic view of digestion, absorption, and metabolism. Carotenoids require fat for optimal absorption, and once formed into vitA, the subsequent physiological processes such as immune system development, growth, and signaling all require adequate levels of complete protein and energy. In addition to the lack of fat, sweet potatoes are also deficient in tryptophan (approximately 0.7% total protein) and are low in other essential amino acids such as histidine and methionine [15,16]. Peanuts are also low in histidine and methionine [17]; therefore, in addition to including a fat source with OFSP, we also included chickpeas to compliment the limiting amino acids [18].

The purpose of the present study was to first identify the optimal ratio of OFSP, peanut paste, and chickpeas that would provide (1) sufficient β-carotene, (2) optimal calories, and (3) complete protein. We first used nutrition software to screen various formulations, followed by mixing, packaging, sterilization, and finally validation of nutritional content. We hypothesized that only three ingredients would be necessary to create a vitA-rich product that provides the necessary components to increase bioavailability, sufficient calories, and complete protein.

## 2. Materials and Methods

### 2.1. Initial Product Design

The initial ingredient formulations were developed using The Food Processor Nutrition and Fitness Software (esha Research, version 11.3.285). The ingredient titles used from this software were Sweet Potatoes, baked in skin, w/ salt, mashed (USDA, ESHA code-5994); Peanut butter, creamy (USDA, ESHA code-4627); and Chickpeas, canned, drained (USDA, ESHA code-38880). Product formulations were developed and tested by varying the percentage of sweet potato, peanut butter, and chickpea, in a 250 g serving. The final product formulation was determined by comparing the nutrient content of each of the tested formulations with RDA reference values. The nutrients analyzed and reference values included total energy (>300 kilocalories), total vitamin A (>900 µg RAE), and an amino acid scoring pattern that met the recommended amino acid scoring pattern for all essential amino acids. Total protein, fat, iron, and zinc were also observed. The nutrient content of the test formulations is shown in Table 1. 

### 2.2. Raw Ingredient Processing

Mississippi-grown raw whole sweet potatoes (Edmuondson Farms, MS., Pack Date: 2 October 2018), Crazy Richard’s 100% natural creamy peanut butter, and Goya canned whole chickpeas were all purchased from a local grocery store in Athens, Georgia. Whole sweet potatoes were washed, sliced into 1 inch disks, and steamed at 95 °C for 10 min. With the skins on, the steamed potato slices were processed into a puree. The jar of peanut butter was stirred to ensure complete mixing. Goya canned whole chickpeas were washed and the skins were removed. Each sample was made in a 250 g serving size, which included 150 g sweet potato puree, 12.5 g of peanut butter, and 87.5 g of whole chickpeas. The ingredients were all weighed, combined in a bowl, and stirred together.

### 2.3. Packaging and Sterilization

One 250 g sample of the product was placed in multiple 50 mL sterile centrifuge tubes. We pipetted 2 mL of glycerol on top of each sample, which was then capped and immediately placed in a −80 °C freezer to protect the β-carotene from oxidation. All other samples were placed into flexible tri-laminate three sealed 4.75″ × 7.25″ flat retort pouches and heat-sealed. Each pouch was filled with a single 250 g sample. The food sample was at room temperature (20–25 °C) when placed into the pouches. Heat sterilization was performed in a Steritort Rotary Sterilizer and was based on a 12 D_121 °C_ reduction, targeting *Clostridium botulinum* [19]. A D_121 °C_ value of 0.2 and a z-value of 10 °C were used to calculate lethality [20]. Following processing, pouches were stored at −80 °C.

### 2.4. Macronutrients, Iron, and Zinc Analysis

Samples were sent to Merieux NutriSciences (Gainesville, FL, USA) in triplicate for chemical nutrient analysis of macronutrients (total calories, total fat, and total protein) and specific amino acids including histidine, isoleucine, leucine, lysine, methionine, phenylalanine, threonine, and valine. Iron and zinc were also chemically tested. Total calories were calculated from total ash (AOAC 925.51A), carbohydrates (calculation), fat (AOAC 933.05), moisture (CRA MOIST.04), and protein (AOAC 992.23). Essential amino acids were measured using the USDA MSS2 (1993) method. Iron and zinc were both measured using AOAC 984.27 methods.

### 2.5. β-Carotene Analysis

The β-carotene content of the raw sweet potatoes (with skin), the unsterilized final product, and the sterilized final product were analyzed in triplicate. All extraction processes were performed under yellow light. All samples were homogenized and 100 mg of the product was placed in 15 mL FalconTubes and mixed with 1 mL of distilled water and 100 µL of trans-β-apo-8′-carotenal, and chilled. Three extractions were completed on each, using chilled acetone as the solvent for the first two extractions and methyl tert-butyl ether (MTBE) as the final extraction solvent [21]. The sample was mixed with the appropriate extraction solvent, vortexed, chilled, and then centrifuged. The top acetone and MTBE layers were removed following each extraction, placed in another 15 mL FalconTube and then placed under nitrogen to dry [22]. After drying, 2 mL of ethyl acetate:methanol, in a 1 to 4 ratio (400 µL ethyl acetate: 1600 µL methanol), was added to the tubes and sonicated. This liquid was syringed through 0.45 mm filters and placed in vials for HPLC analysis as previously described [23]. Extraction recovery was found to be 80%, as determined by using trans-β-apo-8′-carotenal spiked into samples. Reported values were adjusted based on individual sample recoveries.

### 2.6. Statistical Analysis

Percent difference ((actual – theoretical)/(theoretical) × 100)) was used to compare the predicted to the chemical nutrient content of the final product [24]. A paired *t*-test was used to test for statistical significance of β-carotene content before and after sterilization; a priori α was set at *p* ≤ 0.05.

## 3. Results

### 3.1. Total Energy

All the formulations provided at least 300 kcal in a 250 g serving size and energy ranged from 334 to 886 kcal per 250 g. The total vitamin A content of the tested formulations ranged from 722 to 1802 μg RAE. The only formulations that did not meet the target level of 900 μg RAE per 250 g were the two formulations that contained 30% sweet potato. From the estimated formulations, 40% (100 g) was the minimum amount of sweet potato needed in the final product to meet the vitamin A target level. All nine of the essential amino acids were present in all ingredient formulations. However, when comparing the amino acid content with the reference amino acid scoring pattern, the only formulations that met 100% of the recommended amino acid pattern were the 60/10/30, 60/5/35, and 30/20/50 formulations (Table 2). Between these formulations, the lysine and sulfur amino acid scoring patterns were greatest in the 60% sweet potato, 5% peanut butter, and 35% chickpea formulation (Supplementary Table S1). The formulation of 60% sweet potatoes, 5% peanut butter, and 35% chickpeas met all the target nutrient levels per 250 g serving, and was used to make the final food product (Table 1). The physical and sensory characteristics were published previously [25]; however, in brief, the final product resulted in a seemingly heterogenous paste product. The color was bright orange. The whole chickpeas distributed throughout provided a chunky texture to the smooth sweet potato and peanut butter base. The average pH was 5.6.

### 3.2. Macronutrients, Iron, and Zinc Analysis

All nutrients tested were within 10% of the predicted values except for total fat, zinc, and methionine (Table 2). All the predicted values were higher than the actual content expect for histidine, isoleucine, leucine, and phenylalanine. The final energy content in this product was 306 kilocalories, which met the target level. It is important to note that the nutrient composition provides anywhere from 10–30% of daily energy needs based on sex and age (Supplemental Table S2). All the essential amino acids met these recommended patterns except methionine + cysteine (cysteine was not chemically tested) (Table 3). As with total energy, the total protein provided by 250 g ranged from 21–91% of RDA (Supplemental Table S3).

### 3.3. β-Carotene Analysis

The β-carotene content of the raw sweet potato used to produce the final product contained 8180 ± 66 µg of β-carotene per 100 g of fresh weight of sweet potato compared with the predicted value of 17,296 µg of β-carotene per 100 g. There was 8579 ± 213 µg of β-carotene (715 ± 18 µg RAE) in 250 g of the unsterilized product and 5893 ± 135 µg of total β-carotene (491 ± 11 µg RAE) in the sterilized food product (Table 4). The unsterilized and sterilized products were statistically different (*p* = 0.02), with a 31% decrease in β-carotene content in the final sterilized product in part due to water gain and potential oxidative loss. Compared with the theoretical values obtained from the nutrient database, the β-carotene content in the unsterilized food product was 50% lower and the final sterilized food product was 66%. Additionally, the final sterilized product was the only sample that contained both trans-β-carotene (5297 ± 124 µg) and measurable amounts of individual cis-isomers (596 ± 12 µg).

## 4. Discussion

The primary goal of this study was to develop a simple, plant-based food product that provides sufficient amounts of β-carotene, total energy, and protein. Using a nutrient database to develop the initial formulation was useful in this project to produce a product that not only contains sufficient amounts of vitamin A, as pro-vitamin A-carotene, but is also balanced in macronutrients. Since each of the ingredients used in this product contain a unique combination of the target nutrients (energy, amino acids, and vitamin A), changing the amount of one ingredient in the final product changed the composition of these three nutrients. By testing multiple variations, a final formulation that theoretically provides the recommended daily levels of vitamin A, >300 kilocalories, and contains all the essential amino acids was obtained. 

This is a novel approach for developing interventions to treat nutrient deficiencies. When determining nutrient composition, amount, and combinations, multiple co-dependent attributes must be considered while minimizing excess micro- and macro-nutrients, while considering nutrient interactions. The present study was designed to determine if a theoretical composition could be designed, and then we confirmed the nutrient composition within the final product as a proof-of-concept. Although nutrient databases are only estimates, this type of modeling can still be useful in the initial stage of developing a balanced food product and should be used when considering product design for combating undernutrition in a sustainable manner.

Chemical nutrient analysis was essential in this work to validate that the estimates provided from the nutrient database were representative of what was in the actual food product. Previous studies comparing nutrient databases with actual nutrient content of food products showed that most estimated nutrient values are within 10–20% of actual [26,27,28]. In this current project, all the nutrients tested, except for total β-carotene, were within these ranges. All nutrients except for phenylalanine, leucine, isoleucine, and histidine were lower in the actual food product compared with the nutrient database. Previous work has also shown this to be true [29,30]. An important point to consider is that the OFSPs being used in Africa are higher in β-carotene than the commercially available varieties that were obtained for the present studies from U.S. supermarkets [31,32,33]. As such, we anticipate that regions where OFSP is available can use the formulation provided in the present study and expect significantly higher levels than those observed herein.

The final calorie content in this product was 306 kcal per 250 g serving. Although this was 8% less that what was predicted, it still met the target value. This 306 kcal provides 10% to 20% of daily energy needs for adults and over 30% for young children (Supplementary Table S2). In addition to undernutrition, both overnutrition and excessive energy intake is also becoming more prevalent worldwide [1]. Thus, this food product allows for adequate energy to serve as a single meal replacement, but not in excess amounts that can contribute to the problem of overnutrition.

The total fat content was tested to show that fat was available in the final product in sufficient levels that have previously been reported to enhance bioavailability of β-carotene. The final fat content of 7.4 g/serving allows for increased bioavailability of β-carotene. Although the amount of fat needed to increase absorption varies among food sources, as low as 2.4 g of fat was shown to sufficiently enhance the bioavailability of β-carotene in food [34]. Goltz et al. also documented that 3 g of MUFA rich fats has the ability to significantly enhance carotenoid absorption from a single meal in humans [13]. Additionally, 7.4 g of fat is equivalent to 67 calories/serving, which is 22% of the total calories in this product. This percentage of fat is in line with macronutrient distribution ranges that recommend 20–30% of daily calorie needs come from fat. Overall, this suggests that this food product can be incorporated into a well-balanced meal plan.

The total protein content did not differ between the predicted and actual, containing 12 g/serving. This provides 20% to 25% of daily protein needs for adults, around 60% for young children, and over 90% for infants (Supplementary Table S3). Consuming 100% of total daily protein needs in one serving is not recommended because the maximum anabolic use of protein at one time has been shown to be around 25 g [35]. Thus, it is more beneficial to incorporate protein-rich foods throughout the day, which can be provided with a single serving of this product.

In order for protein synthesis to occur properly in the body, not only must total protein needs be met, but also each essential amino acid must be consumed in appropriate amounts. If one or more essential amino acids is not available in the body, protein synthesis will not be able to continue and all other amino acids will not be able to be used. In foods, the amino acid that is provided in the lowest amount needed for protein synthesis to continue is referred to as the limiting amino acid. To determine the limiting amino acid, an amino acid scoring system was developed by the Food and Nutrition Board/Institute of Medicine to compare protein quality among food sources. The reference amino acid scoring pattern was determined by taking the requirement of each essential amino acid and dividing it by total protein needs [36].

Lysine is one of the most common limiting amino acids in regions where protein deficiency is common [37]. This is due to the high percentage of protein consumed from cereal grains, which are limiting in lysine. In order to provide enough amounts of lysine in this product, two legumes, peanuts and chickpeas, were combined. Peanuts are richer in total lysine, 640 mg/100 g, compared with 480 mg/100 g in chickpeas. However, the ratio of lysine per gram of protein is 69 in chickpeas and only 29 in peanuts [38]. Therefore, by incorporating peanuts, which are high in lysine and total protein, with a secondary lysine-rich source, the total lysine content was able to meet the reference amino acid scoring pattern.

The vitamin A content in this final processed food product was significantly lower than the predicted value from the nutrient database. A large reason for this difference can be attributed to the large variation in β-carotene content in raw sweet potatoes. Previous work reported values as low as 111 µg/100 g and up to 7984 µg/100 g fresh weight among varieties [39]. Additional reports also found wide variations in light orange to dark orange varieties, reporting values from 2980 to 22,600 µg/100 g [40]. Bengstsson et al. (2008) observed β-carotene content of seven OFSP varieties and found β-carotene content to be between 3732 and 9648 µg/100 g [41]. Very high β-carotene content has also been shown, with varieties containing 15,000 µg/100 g [42] to 28,100 µg/100 g [43].

Another potential cause for lower than anticipated levels of β-carotene could be our processing techniques. We used care and caution when processing the raw materials, yet once the cell wall is broken, the carotenoids are exposed to air and oxidative degradation can begin. This is likely to have contributed to the reduction in the amounts in the product. Additionally, our thermal processing technique was probably excessive and might be possible to be reduced in terms of time and/or temperature. The prolonged thermal processing may have led to further degradation and loss of β-carotene during processing. To overcome these issues, using a starting material with higher carotenoid content, reducing exposure to air, and optimizing thermal processing can be used to minimize processing-associated loss and improve the total β-carotene content of the final product.

Overall, a single nutrient-rich product was able to be produced from three widely available crops, which could be used to address both macronutrient and vitamin A deficiencies. The total energy content is an appropriate amount for a single meal. Additionally, by combining two protein-rich food sources, all the essential amino acids were provided in appropriate ratios, which makes this a protein-rich plant-based source. The vitamin A content in the final product was significantly lower than predicted, but still provides 100% of the daily needs for young children, and half of daily needs for adults. Additionally, the presence of fat can allow for the β-carotene that is in the product to be more bioavailable for individuals, enhancing the impact of these products in combating vitamin A deficiencies. By incorporating this dish into one or two meals per day, daily vitamin A needs can be met, as well as an appropriate amount of energy and all essential amino acids can be provided.

## Figures and Tables

**Table 1:** Nutrient content of product formulations using The Food Processor Nutrition and Fitness Software.

Ratio of Sweet Potato: Peanut Butter: Whole Chickpeas	75:12.5:12.5	60:30:10	60:10:30	60:5:35	50:30:20	50:20:30	40:40:20	40:20:40	40:50:10	30:20:50	30:50:20
Total Calories (kcal)	403	621	391	334	633	518	760	530	874	542	886
Total protein (g)	13	21	14	12	23	19	28	20	32	21	33
Total fat (g)	17	39	15	9	40	28	53	29	65	29	66
Total Vitamin A (μg RAE)	1802	1442	1442	1442	1202	1202	962	962	962	722	722
β-carotene Equivalents (μg)	21620	17296	17296	17296	14413	14413	11531	11531	11531	8648	8648
Iron (mg)	2.17	2.61	2.27	2.19	2.70	2.53	2.96	2.63	3.13	2.72	3.23
Zinc (mg)	1.58	2.52	1.58	1.34	2.60	2.13	3.14	2.20	3.61	2.28	3.69
Histidine (mg)	300	500	340	290	540	460	660	500	740	530	780
Isoleucine (mg)	410	620	480	440	670	610	800	660	870	720	930
Leucine (mg)	830	1390	920	800	1490	1250	1820	1350	2060	1450	2160
Lysine (mg)	510	720	640	620	820	780	960	880	1000	980	1100
Methionine (mg)	180	270	190	170	280	240	330	350	370	270	390
Cysteine (mg)	150	230	170	150	240	210	290	230	320	250	340
Methionine + Cysteine (mg)	330	500	360	320	520	450	620	480	690	520	730
Phenylalanine (mg)	690	1100	740	640	1180	990	1430	1060	1620	1130	1690
Phenylalanine + Tyrosine (mg)	1070	1800	1130	960	1910	1570	2340	1670	2680	1770	2790
Threonine (mg)	440	600	480	450	640	580	730	620	790	660	830
Tryptophan (mg)	160	240	170	150	250	210	290	220	330	220	340
Valine (mg)	530	790	570	520	840	730	990	780	1100	820	1150

**Table 2:** Comparison of predicted versus actual nutrient content of final formulation after sterilization.

	Predicted	Actual	% Difference
Total calories (kcal)	334	306 ± 4	−8.4%
Protein (g)	12.0	11.8 ± 0.1	−1.1%
Fat (g)	9.0	7.4 ± 0.2	−18.2%
Iron (mg)	2.2	2.0 ± 0.05	−9.5%
Zinc (mg)	1.4	1.1 ± 0.09	−16.1%
Histidine (mg)	290	292 ± 0.01	0.57%
Isoleucine (mg)	440	450 ± 0.00	2.3%
Leucine (mg)	800	825 ± 0.03	3.1%
Lysine (mg)	630	575 ± 0.03	−8.0%
Methionine (mg)	180	150 ± 0.00	−14.3%
Phenylalanine (mg)	650	675 ± 0.03	5.5%
Threonine (mg)	450	442 ± 0.01	−1.9%
Valine (mg)	520	492 ± 0.01	−5.5%

Values obtained from The Food Processor Nutrition and Fitness Software (2016). Actual vitamin A content was obtained from the average of triplicate samples after sterilization.

**Table 3:** Comparison of the actual amino acid pattern to a reference scoring pattern.

	Reference Amino Acid Scoring Pattern (mg/g)	Amino Acid Pattern in Food Product (mg/g)	% of Reference Amino Acid Scoring Pattern
Histidine	15	24.7	165%
Isoleucine	30	38.1	127%
Leucine	59	69.9	118%
Lysine	45	48.7	108%
Methionine + Cysteine	22	12.7 ^a^	58%
Phenylalanine + Tyrosine	38	57.2 ^b^	151%
Threonine	23	37.5	163%
Valine	39	41.7	107%

Reference values obtained from the World Health Organization and United Nations University, 2007. Amino acid pattern determined by taking the content of each amino acid from chemical analysis divided by total protein. ^a^ Amino acid scoring pattern only includes the methionine present. ^b^ Amino acid scoring pattern only includes the phenylalanine present.

**Table 4:** Comparison of predicted β-carotene versus actual β-carotene content in the unsterilized and sterilized products.

	Predicted (µg)	Actual (µg)	% Difference
Unsterilized final product	17,296 (1442) ^a^	8579 +/− 213 (715) ^b^	−50%
Sterilized final product	17,296 (1442) ^a^	5893 +/− 135 (491) ^c^	−66%

Values with different letters indicate significant difference at *p* < 0.05. Actual vitamin A content was the average of triplicate samples adjusted for percent loss.

## Data Availability

The data presented in this study are available on request from the corresponding author.

## Supplementary Materials

The following are available online at https://www.mdpi.com/article/10.3390/foods10051019/s1, Table S1: Essential amino acid patterns of formulations compared to reference amino acid scoring pattern requirements, Table S2: The percentage of daily energy requirements provided from 250 g, Table S3: The percentage of the RDA for total protein provided from 250 g, Table S4: The percentage of the RDA of vitamin A (µg RAE) provided per 250 g.
