# Supplementary material for: Design and Nutrient Analysis of a Carotenoid-Rich Food Product to Address Vitamin A and Protein Deficiency
Source: Foods. 2021 May 7;10(5):1019. doi: 10.3390/foods10051019 (PMC8151009; doi:10.3390/foods10051019)
Supplement: Supplementary file 1 [file foods-10-01019-s001.zip › foods-1208918-supplementary.pdf]

Table S1. Essential amino acid patterns of formulations compared to reference amino acid scoring pattern requirements.

	Score	75:12.5:12.5	60:30:10	60:10:30	60:5:35	50:30:20	50:20:30	40:40:20	40:20:40	40:50:10	30:20:50	30:50:20
Histidine	15	23	23	25	24	24	24	24	25	23	25	24
Isoleucine	30	32	29	35	37	30	32	29	33	28	34	28
Leucine	59	64	65	66	67	66	66	66	67	65	68	66
Lysine	45	39	34	46	52	36	41	35	44	32	46	34
Met + Cys[b]	22	26	23	26	27	23	24	22	24	22	24	22
Phe + Tyr[c]	38	83	84	82	80	84	83	84	83	85	83	85
Threonine	23	34	28	35	38	28	31	26	31	25	31	25
Tryptophan	6	12	11	12	13	11	11	10	11	11	10	10
Valine	39	41	37	41	43	37	39	36	39	35	39	35

Table S2. The percentage of daily energy requirements provided from 250 grams.

Age Group (years old)	Male	Female
1-3	22-31%	22-31%
4-7	17-26%	17-26%
8-12	13-22%	14-22%
13-18	10-15%	13-19%
19-50	10-13%	13-17%
>50	11-15%	15-19%

Table S3. The percentage of the RDA for total protein provided from 250 grams.

Age Range (years)	Percent of RDA
1-3	91%
4-8	62%
9-13	35%
14-18	23-26%
19-50	21-26%
>50	21-26%

Supplemental Table 4. The percentage of the RDA of vitamin A (μg RAE) provided per 250 grams.

Age Group (years old)	% of RDA provided
0-1	98-123%
1-3	163%
4-8	123%
9-13	82%
14-18	55-70%
19-30	55-70%
31-70	55-70%
>70	55-70%
Pregnant	65%
Lactating	41%
